# Financial Barriers to Success: Opening the Discussion of the Financial Burdens and Graduate Student Experiences in Bioarchaeology and Forensic Anthropology

**DOI:** 10.1002/ajpa.70182

**Published:** 2025-12-12

**Authors:** Abigail Elaine Houkes, Laura Cirillo

**Affiliations:** ^1^ Department of Anthropology University of Illinois Urbana‐Champaign Urbana USA; ^2^ SNA International Alexandria USA

**Keywords:** biological anthropology, equity, financial barriers, graduate students, institutional reform

## Abstract

**Objectives:**

This study examines the financial barriers faced by graduate students in bioarchaeology and forensic anthropology, addressing a critical gap by incorporating recent perspectives. Prior research has highlighted financial inequities within the field, yet few studies focus on burdens impacting students' well‐being, academic success, and career paths. This research aims to amplify student voices and identify actionable, student‐centered solutions to alleviate financial strain and support retention.

**Materials and Methods:**

A 29‐question anonymous survey was distributed to current and recent biological anthropology graduate students (*n* = 103) across the United States. Questions covered various financial factors, including institutional costs, students' lived experiences with strain, and career advancement expenses. Responses were analyzed using descriptive statistics for quantitative data and thematic coding for qualitative responses.

**Results:**

The survey revealed that nearly all participants depend on some form of financial aid (assistantships, grants, fellowships, or scholarships), with many reporting insufficient stipends, unmet living costs, and a need for external employment. Over half‐expressed concerns about financial impacts on career opportunities, with many using loans or credit to cover essential costs like conferences, travel, and program fees. Financial strain significantly impacted well‐being, with 58% considering leaving the field due to financial pressures.

**Discussion:**

The findings underscore the urgent need for institutional reforms to ensure livable wages, transparent funding, and professional development support. Addressing these barriers is essential for retaining diverse talent and fostering a sustainable future in bioarchaeology and forensic anthropology. This study advocates practical solutions to reduce financial inequity and promote a more inclusive academic environment.

## 1 Introduction

The conversation exploring the financial considerations that college and graduate students face has garnered considerable attention in social science literature. Previous research has addressed these concerns from multiple angles, including private institutions (Moore et al. 2021), regional focus (Güler et al. 2024; Laframboise et al. 2023), specific demographic populations (Donner 2021; Lasch 2018; Moyer et al. 1999), and specific fields of study (Szkody et al. 2022). Graduate students face a range of persistent and pervasive financial concerns. The costs of tuition and fees from institutions, cost of living, care necessities, and the often‐unanticipated burden of networking and career advancement opportunities (e.g., travel expenses and professional membership dues) all factor into student financial decisions (Cantwell‐Chavez and Rowland 2022; Hawke and Hulse 2023). Students supplement their income with additional employment, governmental assistance, and debt or risk not completing their graduate program (Dunyak 2018; Pyne and Grodsky 2020; Strayhorn 2010).

Within the United States, graduate students are often employed through research or teaching appointments where students may receive a monthly stipend and a tuition waiver (full or partial). Still, for most graduate students, financial situations are tied to factors such as grants, scholarships, loans, and familial support (Johnson et al. 2020). Students often feel compelled to seek additional work; however, some universities stipulate that graduate students cannot work for external employers, exceed a certain percentage of work, or are prohibited from working entirely (Delgado et al. 2024; Johnson et al. 2020). Within biological anthropology, graduate students frequently engage in a range of unpaid, underpaid, or deficit‐funded activities to remain competitive post‐graduation. In this context, unpaid labor is defined as labor undertaken without direct monetary compensation or requiring the student to incur personal costs for activities that are not considered volunteer work. Volunteer work is differentiated as services that a paid person does not normally perform and not simply the willingness of the participant. The scope of activities that constitute unpaid labor discussed in this work is limited to those related to the student's academic and professional experience. These activities include, but are not limited to, unpaid additional time when task timelines are underestimated (e.g., conducting fieldwork and casework beyond contracted assistantship hours), travel and attendance at academic or professional events directly related to the graduate education at personal cost (e.g., presenting at conferences), and time‐intensive tasks that are expected to be completed outside of work hours resulting in a professional product (e.g., publishing research). Activities that fall under the category of unpaid labor are often framed as essential components of graduate training through professional development but represent a significant financial burden beyond the “sticker price” of programs. This burden is compounded for those facing acute economic insecurity, as their capacity to participate in unpaid professional activities is severely limited.

Unpaid labor in this context can also encompass what has been described as aspirational labor—work undertaken in the hope of future career advancement without any guarantee of financial return (Grant‐Smith and McDonald 2018). Conference presentations and research collaborations are often accepted with the indirect expectation of compensation through access to subsequent paid employment. These “opportunities” (consider the implications of this common term) often require out‐of‐pocket expenses for travel, lodging, registration fees, and materials. These costs can be prohibitive and may compel students to forgo paid employment or accrue additional debt to participate.

When all forms of uncompensated and undercompensated labor are considered together, graduate students may work up to a combined 60–70 h per week, and the potential inability to work outside of the university setting, which can significantly diminish their effective hourly stipends and contribute to heightened stress and mental health challenges (Delgado et al. 2024). While these activities can provide professional benefits that extend beyond direct pay by potentially improving employability, any assumption that all students can participate presumes a baseline level of financial security. Adequate baseline funding and institutional support are essential to ensure that participation in these career‐advancing activities does not result in disproportionate hardship. Without such support, the expectation that students will independently finance these experiences risks reproducing inequities in professional advancement and well‐being. Ongoing financial pressures have been linked to a two‐ to six‐fold increase in stress and psychological distress among graduate students compared to the general population (Bekkouche et al. 2022), underscoring the urgency of addressing these structural challenges.

While there has been a call for promoting Diversity, Equity, and Inclusion (DEI) broadly within the discipline of biological anthropology (Antón et al. 2018; Erhart and Spradley 2022; Fuentes et al. 2019; Malhi et al. 2019; Spiros et al. 2022; Tallman and Bird 2022; Tallman et al. 2022; Turner et al. 2018), and some discussion of the complexities of socioeconomic status affecting undergraduate and graduate students, the broad scope of previous research precludes the depth of discussion on financial burden by introducing multiple topics from multiple perspectives. Because of the established financial strain affecting graduate students, a better understanding of their financial experiences will help identify primary concerns and areas for response.

This research aims to explore the common financial experiences among graduate students in bioarchaeology and forensic anthropology programs by analyzing survey data of student perspectives, with two primary objectives: (1) the inclusion of graduate student voices in the ongoing conversations concerning finances through direct graduate student engagement, and (2) provide data to indicate student‐centered solutions to the financial burden on graduate students with the goal of increased welfare and retention within these fields. Our research models an inclusive framework by allowing underrepresented graduate student voices to be heard and highlighting results derived directly from the perspectives of graduate students. This research aims to emphasize solutions based on graduate student experiences by identifying the needs of survey participants and demonstrating which aspects of finances are of the most immediate concern, in order to help institutions focus their efforts and maximize support.

## 2 Materials and Methods

An anonymous survey was constructed and distributed online through a forensic anthropology listserv and multiple Facebook groups targeting multiple subfields (University of Illinois Urbana‐Champaign IRB #24328). The sample population for this study consisted of current and recent graduate students (within the past 5 years) in biological anthropology. The study was limited to United States residents attending university domestically to avoid the unique limitations of student visas.

The survey included 29 multiple‐choice and open‐ended field questions designed to gather quantitative and qualitative data on participant demographics and experiences (Supporting Information Table A). Quantitative questions focused on background demographic information, financial aid (assistantships, grants, scholarships, and fellowships), institutional support, and perceived areas of impact. Qualitative questions focused on personal experiences and effects on mental health, providing an opportunity for participants to elaborate on the quantitative questions. Some questions initiated conversations on subjects that were previously identified as significant when discussing student finances in other contexts, including mental health (Armstrong et al. 2024), attrition (Strayhorn 2010), and feelings of isolation (Moore et al. 2021). The goal of the survey was to provide graduate students an outlet to voice their thoughts regarding finances in graduate school while collecting comparative data, providing additional insight into the interpretation of the data collected. The survey was conducted anonymously to alleviate potential stress stemming from the taboo of discussing finances and to encourage the sharing of information without fear of repercussions.

Participants were not asked about their personal demographics (e.g., gender, age, etc.) to ensure anonymity and avoid the risk of misattribution and overgeneralization of demographic data we too often see in survey interpretation. Demographic data based on artificially rigid categorization of identity further compounds the potential error. The lack of diversity in biological anthropology has been acknowledged (Antón et al. 2018), so additional questions may not provide enough data to elicit meaningful patterns, despite acknowledgment that individuals from diverse backgrounds often face systemic barriers to higher education funding.

Participants were also not asked to specify an institution for two reasons. First, the authors were focused on understanding more general, shared experiences and were concerned that identifying specific institutions would provide an opportunity for other institutions to more easily dismiss the findings of this research because they were not directly implicated. The authors experienced instances of this while designing the research and would like to stress the communal responsibility that needs to be taken. Second, the authors wanted participants to feel safe, given the sensitive nature of the topic and, unfortunately, the very real fear of retaliation. The collected demographic data were included to ensure a sufficient variety of respondents through diversity of region (allowing the authors to track the proportions of responses from each region to prevent over‐representation of any particular institution), subfield, and degree level, where no single category should represent > 50% of participants. Rather than seeking correlations with these variables, these data ensure the patterns in responses are widely applicable to graduate students in forensic anthropology and bioarchaeology. Quantitative data were analyzed through descriptive statistics of the sample population of respondents. Open‐ended questions were analyzed using qualitative coding methods. Open coding was employed to identify patterns and themes in the responses, followed by more focused coding to refine these themes.

## 3 Results

### 3.1 Academic Demographics and Graduate Funding

After excluding incomplete surveys, 103 responses remained. Forensic anthropology was the most represented subfield (57%), followed by bioarchaeology (26%) and human biology (10%); all other subfields represented < 7% of total responses. For the quantitative portion of this analysis, participants in fields representing < 10% of total responses were removed (adjusted sample size of 96 responses). Most respondents reported being current Ph.D. students with an M.A. (32%), followed by recent Ph.D. graduates (within 5 years) (24%) and recent M.A. graduates (20%), with a range of time spent within the program reported (Table 1). Participants were asked to indicate their graduate program location among four territories in the United States. Each territory represented 10%–38% of the participants, indicating a relatively diverse representation of programs in the results. Although these demographic groups are not equally weighted, the diversity of responses was satisfactory for the scope of this preliminary study.

**TABLE 1:** Current education stage and program length of surveyed students in Forensic Anthropology, Bioarchaeology, and Human Biology (*n* = 96).

Current Education Stage (Years In Stage)	*n*
Current Master's student	15
< 1 year	1
1 year	8
2 year	2
3 year	3
4 year	1
5 year	0
6+ year	0
Recent Master's graduate (no PhD education)	19
Current PhD student (with Master's)	30
< 1 year	1
1 year	5
2 year	3
3 year	10
4 year	5
5 year	4
6+ year	2
Current PhD student (without Master's)	8
< 1 year	1
1 year	1
2 year	0
3 year	0
4 year	2
5 year	2
6+ year	2
Recent PhD graduate	24

Almost all surveyed graduate students (95%) reported participation in some form of financial support program, including scholarships, grants, assistantships, student loans, and so forth. When excluding loans, most participants (43%) reported receiving $10,000 – $50,000 in scholarships, grants, or assistantships across the entire duration of their graduate studies, while very few (8%) reported receiving < $1000 over that period (Table 2). External grant funding was reported by 28% of participants.

**TABLE 2:** Reported cumulative funding received during graduate studies (excluding tuition waivers) with average number of years in current program for active students and percent of graduated respondents reporting that final amount.

Amount	*n*	Average Year in Program (rounded; excluding graduates)	% of Graduated respondents
Amount less than $1000	8	1	14
Amount between $1000 and $5000	10	2	7
Amount between $5000 and $10,000	12	3	16
Amount between $10,000 and $50,000	41	3	35
Amount between $50,000 and $90,000	14	4	12
Amount over $90,000	11	6	16

Graduate program funding often includes a partial or full tuition waiver and possible stipend in exchange for graduate student labor under an assistantship (e.g., teaching assistant, graduate assistant, etc.). Most participants (82%) had participated in an assistantship (average of 19 h per week). Outside of assistantships, 25% of graduate students have worked a job within the university (average of 17 h per week). Despite a larger number of respondents, reporting employment under an assistantship, partial to full tuition waivers were only reported by 76% of participants, indicating some students did not receive any form of tuition waiver. Of the respondents receiving a waiver, 46% received one for the entire length of study.

### 3.2 Financial Strain and Insecurity

The results indicate a staggering financial strain felt by students in forensic and bioarchaeology graduate programs. While enrolled in graduate school, 50% of participants have utilized government aid to afford health care, groceries, or utility bills. Additionally, 29% of surveyed students used a food pantry or other free services to access food or other necessities while enrolled in graduate school.

These financial concerns negatively impacted the academic experiences of the students. 49% of participants were concerned about attending on‐campus events (e.g., work, classes, etc.) due to the cost of transportation. Only 18% indicated their graduate program provided a livable wage (as defined by the minimum amount of financial assistance to cover living costs such as rent, food, utilities, and so forth, without the use of loans or credit). The survey found that 81% of participants used credit cards or loans to cover costs, expecting to repay them later. Common expenses prompting the use of credit or loans included graduate fees (68%), conferences (65%), relocation (59%), transportation (55%), and professional attire (53%) (Table 3).

**TABLE 3:** Experiences garnering debt during graduate school that were paid using a credit card, personal loan, or governmental loan.

Activity	*n*
Graduate School Fees	65
Conference	62
Relocation/Moving Costs	57
Transportation	53
Professional Attire for Networking Opportunity	51
Tuition	41
Research Opportunity	30
Field School	29
Workshop	19
Internship	16
Certification	11

### 3.3 Financial Impact of Career‐Advancing Activities

This study examined their financial burden by comparing out‐of‐pocket costs to compensation for internships, field schools, conferences, and research opportunities, representing activities critical for career advancement (Table 4). Fewer than 10% of participants were paid or incurred no cost for all activities. For conferences, only 2% were paid to attend, and 1% had no cost. Most respondents personally paid 50%–100% of the costs of internships and field schools. Both had the highest N/A responses (54% and 40%), suggesting many did not participate because the perceived value did not justify the cost. Conferences had the highest participation rate, with only 6% reporting no involvement, underscoring their importance in the represented subfields. Of those attending, 29% paid less than half the cost, while 62% paid 50%–100%.

**TABLE 4:** Degree of financial expense by activity.

Activity	*n*						Total
	I was paid to participate	I paid 0%	I paid some (under 50%)	I paid most (over 50%)	I paid 100%	N/A	
Internship	4	7	3	5	**25**	52	96
Field School	4	4	7	5	**37**	38	95
Conference	2	1	28	**38**	21	6	96
Research Opportunity	6	9	**22**	15	19	25	96

*Note:* *Bold indicates the highest non‐N/A value. This represents the most common participant experience.

The survey also asked whether graduate students had forgone activities they considered more than moderately beneficial to their careers due to cost (Table 5). Students evaluated these situations based on two criteria: (1) providing significant improvement not easily gained elsewhere, and (2) without it, a perceived detriment to employment or funding opportunities. Internships had the highest N/A responses (31%), suggesting they may be viewed as less essential in these subfields. Field schools (55%) and conferences (68%) were most often declined due to cost. Conferences had the fewest N/A responses (4%), indicating they are widely valued, yet expense still prevented participation for 68% of students.

**TABLE 5:** Reported opportunities declined or not pursued despite perceived career benefit.

Activity	*n*		
	Yes	No	N/A
Internship	**34**	32	29
Field School	**52**	30	13
Conference	**65**	26	4
Research Opportunity	35	**45**	15

*Note:* *Bold indicates the highest value.

### 3.4 Perceptions of Social Support and Pressure

**FIGURE 1:**
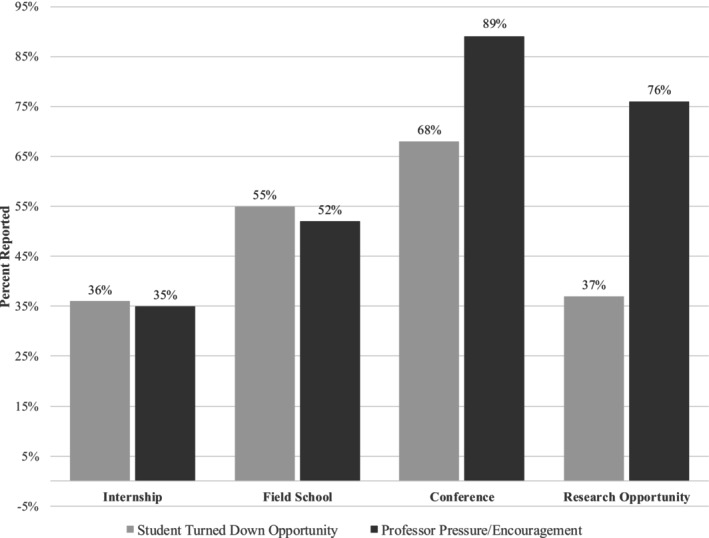
Comparison of student beliefs about beneficial activities versus professor pressure/encouragement.

This survey examined faculty influence on the perceived value of internships, field schools, conferences, and research opportunities (Table 6). Most respondents reported faculty pressure to attend conferences (89%), pursue research (76%), and join field schools (52%). The high expectations, coupled with the financial burden and rates of non‐participation, are notable, as the most encouraged activities carried the highest costs (Figure 1). Many students were reluctant to discuss finances with peers (55%) or advisors (65%), citing social discomfort. Explicit program rules or faculty requests accounted for < 10% of reasons for avoiding such discussions.

**TABLE 6:** Reported pressure and/or encouragement from a supervisor to participate by activity.

Activity	*n*		
	Yes	No	N/A
Internship	34	**37**	25
Field School	**50**	30	16
Conference	**85**	9	2
Research Opportunity	**73**	17	6

*Note:* *Bold indicates the highest value.

When asked if current research on financial inequity among biological anthropology graduate students is sufficient, 48% disagreed and 33% strongly disagreed. Regarding faculty advocacy for graduate student financial support, 34% disagreed, 38% agreed or strongly agreed, and 20% were neutral. Only 8% strongly disagreed, but overall, the results do not indicate a positive trend in perceived faculty support.

The survey also asked whether financial pressures during graduate school had negatively impacted mental health. A total of 59% strongly agreed and 28% agreed, while only 4% disagreed, indicating a widely shared perception of impact. When asked if they had considered leaving biological anthropology due to financial costs, 58% had at least considered or actually left the field.

## 4 Qualitative Findings

The entire sample (*n* = 103) was retained for the qualitative portion of analysis. The authors deemed it important to include the voices of all participants to represent graduate students' interpretation of the survey topics more comprehensively. Coding of the open‐ended response portion of the survey revealed several dominant themes (Supporting Information Table B).

### 4.1 Unanticipated Financial Burden

Across the dataset, an overwhelming financial burden outside of the anticipated costs of a graduate program emerges as the most frequently cited issue. Many respondents struggle with the high cost of living, particularly in major cities where rent and basic expenses surpass the stipends or wages their graduate programs provide. Some mention relying heavily on student loans, adding to the stress of accumulating debt.

### 4.2 Career Uncertainty

The data also reflect considerable career uncertainty, with many respondents questioning the value of their degrees and the feasibility of pursuing long‐term careers in biological anthropology. Several respondents cited job market saturation and limited opportunities as significant concerns. Some individuals expressed regret and frustration over investing time and money into a field that offers little financial reward or job security in return.

### 4.3 Leaving or Considering Leaving the Field

Financial difficulties and job insecurity led some respondents to leave the field while others actively considered it. Those who have already left noted that they found more financially stable careers outside academia, where they earn significantly higher wages without the constant pressure of academic work. Those contemplating leaving the field expressed frustration over the lack of stable employment opportunities and sacrifices with no guaranteed payoff. Others are contemplating temporary breaks, hoping to rebuild their savings before potentially returning to the field. This exodus is often framed as a necessary but painful decision based on a more realistic understanding of career prospects only reached at the end of an academic program.

### 4.4 Familial and External Support

External financial support, particularly from family members or spouses, has been crucial for those who remain in the field. Several respondents mentioned relying on a partner's income or parental assistance to continue their studies, acknowledging that they likely would have left the field without this support. This reliance on community support underscores the broader issue of socioeconomic inequality within biological anthropology.

### 4.5 Mentorship and Guidance

Mentorship played an important role in shaping respondents' financial expectations and career decisions. Some respondents appreciated the transparency and guidance provided by their mentors, who helped them navigate the financial challenges of graduate school. However, others felt their advisors failed to adequately address the realities of the job market and the financial hurdles they would face, leaving them feeling unprepared and unsupported.

### 4.6 Diversity and Access

Respondents highlighted that those from wealthier backgrounds or with familial financial support are better able to take advantage of career‐advancing opportunities that are unpaid/underpaid/cost‐deficient, like field schools, conferences, and internships. This results in a lack of access for students from lower‐income backgrounds, who often cannot afford the costs associated with gaining meaningful experience in the field. The socioeconomic inequities add to feelings of isolation and inadequacy among those without such support. Some respondents feel discouraged watching peers succeed while they struggle to finance necessities.

### 4.7 Stress and Mental Health Impacts

The responses highlight significant stress and mental health challenges stemming from the financial strain and career uncertainty faced by individuals in these fields. Many respondents pointed to financial instability as a primary source of their stress. The burden of student loans and the pressure to fund research and conference participation added to the financial stress, with some respondents expressing fears of falling deeper into debt. Several respondents reported being mentally exhausted from trying to balance multiple jobs, academic coursework, and research, mainly when assistantships or stipends were inadequate. Some felt the weight of this burden affected their academic performance, mental clarity, and overall well‐being.

For those who had left or were considering leaving the field, the decision often came after intense mental and emotional strain. Leaving a field, they had invested so much in caused feelings of guilt, disappointment, and personal failure.

Several respondents noted that the cumulative effect of prolonged financial stress had caused a decline in their mental health. For some, this pressure led to burnout and feelings of hopelessness as they struggled to keep up with the demands of academia. Some described the mental toll of making extreme sacrifices just to stay in the field. Stories of living in cars or skipping meals reflected how respondents were coping with financial strain at the expense of their mental and physical health. Despite the significant mental health challenges, some respondents expressed a sense of resilience, continuing to push forward in the hope that their persistence would eventually lead to a stable career.

### 4.8 Graduate Program Reforms

Respondents offered several recommendations for improving financial support in forensic and bioarchaeology graduate programs. The most common was to increase stipends to a livable wage, as current amounts often fail to cover basic expenses such as rent, food, and healthcare. Many also called for additional internal grants and scholarships to offset the costs of research, fieldwork travel, and conference attendance, which were frequently cited as significant financial burdens. Others recommended that departments facilitate access to external funding by creating centralized resources and providing application assistance. Several respondents suggested that universities should provide affordable or subsidized housing options for graduate students. They also suggested that internships and field schools should offer financial compensation, as many currently do not. Respondents noted the need for professional development grants or funding to allow students to gain experience outside of academia.

There was a strong call for more transparency around funding and career prospects. Respondents called for guaranteed funding packages that cover the entire duration of their graduate studies, especially PhD programs. Many also stressed the need for consistent funding throughout the year, especially during the summer months. Participants in this study want universities to be transparent about total costs and available funding. Many felt that universities should also be more transparent about job market prospects for students, noting that students are often unprepared for the limited job opportunities and low academic wages after completing their degrees.

Some respondents suggested that universities provide comprehensive health insurance. Mental health was another significant concern, and respondents recommended expanding access to mental health services for graduate students, acknowledging the stress and burnout many students experience.

There was a strong call for universities to stop accepting more students than they can fund adequately. Several respondents argued that departments should limit the number of students they admit to those they can fully fund. They felt that programs accepted more students than the department could provide financial support, which leads to inequitable experiences where some students receive funding and others struggle. They suggested smaller cohorts that would allow for fully funded opportunities and more personalized mentorship.

## 5 Discussion

This introductory study underscores the pervasive financial struggles among graduate students, reflecting both individual hardships and systemic challenges. The resulting perceptions and impacts of financial strain elicit discussion of the broader implications of these findings, the contributing structural factors, and the need for a holistic approach to financial reform within graduate programs. Although this is a small‐scale study, several general trends can be reasonably applied to the demands of the broader system of graduate study in biological anthropology.

### 5.1 Financial Challenges as Barriers to Career Advancement

One of the most striking findings from this study is that financial limitations not only affect students' day‐to‐day lives but also create barriers to essential career development opportunities. Survey participants noted that they frequently encountered substantial expenses associated with activities vital for career growth, such as attending conferences, participating in field schools, and networking at professional events. These activities are outside of the “sticker price” of an academic program and represent a major uncalculated expense. The lack of financial support for such activities forces many students to either take on debt or forgo these opportunities, ultimately limiting their professional development and reducing their competitiveness in the job market. Many students enter post‐graduate life with debt accrued from both tuition and the hidden costs associated with academic advancement. These financial strains contribute to a widening gap between students with access to external financial support and those without, creating a cycle of exclusion for economically disadvantaged students.

### 5.2 The Burden of Unpaid Labor and Socioeconomic Inequity

Unpaid labor was another recurrent theme in the data, reflecting a broader issue within academia where graduate students are often expected to engage in “voluntary” work to bolster their credentials. In these fields, unpaid or underpaid work can include tasks like fieldwork, internships, and research collaborations. Although these commitments are often viewed as essential for building a professional network and academic portfolio, the financial demands of this unpaid work are unsustainable and disproportionately affect students from lower‐income backgrounds who may not have external financial support. This undermines DEI efforts within the field beyond admitting diverse students. While programs seemingly promote inclusion by accepting these students, they fail to support them through these unspoken expectations and ultimately set them up to be viewed as non‐competitive compared to students with fewer financial restrictions.

### 5.3 Institutional Gaps in Support and Transparency

Another critical finding from this study is the perceived lack of institutional support and transparency regarding funding, employment prospects, and financial expectations. Despite the shared information through informal channels (e.g., online forums and professional organizations), many students felt the absence of transparent communication directly from academic institutions regarding available funding and anticipated expenses created a sense of financial uncertainty and made it challenging to plan for the financial realities of graduate education.

These patterns are shaped by budget mechanics beyond the control of any single department. Stipends and tuition waivers are typically governed by central graduate colleges or deans' offices, while departments may have limited discretion beyond small internal awards or travel grants. Additionally, overhead rules, grants, or donor terms restrict such money. As a result, some of the remedies proposed by the respondents (e.g., stipend increases, fee waivers) require university‐level action, whereas others (e.g., professional‐development funds, local fee reductions) can be implemented within departments. Recognizing this multi‐level structure clarifies why responses must be tailored by region, institution type, and degree level (Morgan et al. 2022).

To address these issues, institutions must adopt transparent policies that provide students with a realistic picture of available funding and job market conditions, allowing students to make informed financial decisions.

### 5.4 Proposed Solutions and Institutional Reform

A significant portion of graduate students recommended increasing stipend amounts to more accurately reflect local living costs, especially in high‐cost areas. Adjusting stipends to account for inflation and the rising cost of living would alleviate some financial pressure on students, allowing them to focus more fully on their academic responsibilities. These adjustments should consider other concerns in calculating an expected “cost of living” for students, including the undervaluation when students report their expenses, given students already sacrifice vital expenses.

While the desires of the respondents may seem unrealistic, smaller steps can be implemented by faculty and department heads to move toward these goals. One vital step is increasing transparency for both current and prospective graduate students regarding departmental financial structures. As noted above, students want transparency but often don't know the mechanics of department funding enough to make any specific suggestions. As a result, respondents request things like “increased stipends” that may be easily dismissed as uninformed by an administration. Consider the identified areas from a student perspective. These identified needs are where students feel the greatest impact and are placing their priorities.

Transparency should include clear explanations of how funding operates and the mechanisms in place to support students, including discussions of the priorities of departments versus colleges or universities. If students are not given a direct means to advocate for their own needs, they need to understand who their advocates are and what discussions are taking place behind closed doors. This relies on the promotion of and participation in shared governance within the university (Scott 2020), a principle that recognizes the importance of collaborative decision‐making among administration, faculty, and students. Faculty buy‐in to these collective efforts can contribute to more equitable institutional practices for both the financial demands on students and the related workload policies for faculty.

Beyond transparency about graduate programs' finances, faculty and staff can also create supportive spaces for open discussions about finances. Addressing this requires intentional efforts from advisors and faculty to foster respectful, nonjudgmental dialog. This can be achieved by being willing to listen to students and supporting students' calls for change, including supporting graduate student unionization efforts or, in the context of “right to work” states, advocating and supporting “good‐faith bargaining” (Perold and Dirnbach 2025). Although the focus is often within individual departments or colleges, a larger, collective buy‐in is required across the discipline for efforts like raising the national average for stipends for teaching assistantships.

Another frequently proposed solution is expanding internal grants, particularly for career‐building activities such as fieldwork and conference attendance. A practical solution could involve establishing a student professional development fund that is explicitly advertised to donors for these purposes. Clearly defining what activities the fund supports, while maintaining flexibility in its use, would allow students to participate in a wide range of professional opportunities.

For graduate advisors, when possible, prioritizing the incorporation of students' financial needs within grants should be considered. Although not all grants allow stipends, prioritization of this temporary funding can alleviate the financial burden for some students. Although advocating for students needs to take place at higher levels, incremental change will place a lot of the initial burden on individual faculty. With collective buy‐in stemming from individuals modeling advocacy, the shift in culture can progress to shift the burden off students and advisors to the department and university (Hellwig 2024).

It is obvious that departments are not purposefully withholding additional available funding from students. Acknowledging this, there is a strong call for institutions to limit enrollment to the number of students they can adequately support. Accepting more students than a program can financially support creates inequitable experiences where some students have access to funding and others struggle to cover basic expenses. The current model of graduate school enrollment eliminates many highly capable students, potentially squandering department investments.

Limiting admission to funded students would enable more personalized mentorship and a more effective allocation of resources, ultimately fostering a more supportive and sustainable graduate education environment. Knowingly admitting students when funding and mentorship resources are already strained among existing students is an unethical and unsustainable practice. While institutions face complex barriers in funding processes, graduate programs should carefully consider how to allocate resources for students and consider holding such conversations prior to accepting students, rather than the common practice of holding funding meetings after students are accepted. As stated in previous research, such as Rohden et al. (2024), graduate students are more than just a line item in a university budget. Although graduate students are not a financial priority for universities, there is a level of investment that we need to advocate for as a field to sustain or improve our place in science. This advocacy includes changing the perceived punishment of increasing teaching load for advisors that don't meet a certain quota of advisees if funding resources limit the addition of more students. This advocacy should not solely be focused on universities but also consider policy and greater structures that nationally fund our work.

### 5.5 Negative Impacts on the Field

The financial burden of graduate education is often viewed as a personal burden to be met by the student. The broader effects of this culture of economic crisis are less frequently considered, but these perceived personal problems contribute to systemic instability that diminishes the field overall.

A broader concern is the financial contribution to the increased rate of attrition. Many respondents indicated that the heavy financial burdens, combined with limited job stability and low pay post‐graduation, make staying in biological anthropology unsustainable. Qualified students leave these fields for more financially stable career options, impacting talent retention within biological anthropology. Given the competitive and underpaid nature of jobs in these fields, many graduates struggle to achieve financial stability, making it challenging to justify the financial investment required for graduate education in the field. This cost–benefit imbalance ultimately devalues the field as a whole.

The reliance on unpaid/underpaid labor for tasks essential to professional development reinforces an inequitable system where only those with external financial support or fewer financial obligations can fully participate. Perpetuating exploitative labor models in graduate school devalues jobs in forensic anthropology and bioarchaeology by normalizing low or unpaid work, reducing incentives to improve compensation, and limiting job stability. Exploitative labor models often lead to career paths with limited financial stability and job security, as many positions, including postdoctoral roles and adjunct teaching posts, are underpaid and short‐term. Graduates may find themselves in a series of temporary, low‐paying jobs, mirroring the financially precarious positions they held as students.

When graduate students are often expected to perform unpaid/underpaid tasks in research and teaching, these activities become devalued in the academic and professional settings of biological anthropology. This normalization creates an expectation that essential work, especially early in one's career, does not merit fair compensation. As a result, entry‐level positions and other roles in the field are often structured around minimal pay or even voluntary work, setting a precedent that is difficult to change. The reliance on low or unpaid graduate labor reduces incentives for institutions and employers in biological anthropology to offer competitive wages or benefits for academic and professional positions. This situation can lead to stagnation in wage growth and job quality, affecting the field's ability to attract and retain skilled professionals beyond opportunities that are deemed entry‐level.

The field is also threatened by reduced diversity and inclusion. Exploitative labor models inherently limit access to the field for individuals from lower‐income or underrepresented backgrounds who cannot afford to work unpaid or low‐wage jobs. This reduction in socioeconomic diversity restricts the variety of perspectives and experiences within the field, which are crucial for advancing innovative research and inclusive practices. Consequently, the devaluation of labor in biological anthropology not only impacts individual job value but also stifles the field's intellectual diversity and progression. This restriction limits the diversity of perspectives, research topics, and approaches within biological anthropology. The field's valuable DEI efforts are thus undermined when financial barriers prevent diverse scholars from participating and thriving.

Addressing financial inequities would raise the baseline for wages, benefits, and working conditions across the field. Fair compensation for stipends, funding, and entry‐level roles sets a precedent for competitive salaries throughout the career pipeline, from postdoctoral researchers to senior faculty. This normalization of fair pay would reduce job insecurity, creating more stable and financially viable careers in forensic anthropology and bioarchaeology and making the field more attractive to emerging scholars. Senior professionals would also benefit from a steady pipeline of talented, diverse junior researchers and collaborators, essential for the discipline's continued growth and evolution.

### 5.6 Limitations and Future Directions

While this exploratory study provides valuable insights into the financial burdens faced by graduate students in forensic anthropology and bioarchaeology, several limitations warrant consideration. The web‐based survey yielded a disproportionate number of respondents from these subfields, likely reflecting the networks through which it was disseminated, including targeted email listservs and social media. This recruitment approach introduces non‐response bias, as individuals outside these networks may have had relevant experiences not captured by the current survey (Hendra and Hill 2018). Additionally, the sample is subject to self‐selection bias, whereby students experiencing greater financial strain may have been motivated to participate (Bethlehem 2010).

Centering this research exclusively on graduate students emphasizes a more complex examination of financial realities. The sample size limits broad interpretation, but the voices represented in this study are nonetheless indispensable. The willingness of graduate students to speak candidly about financial strain, an often stigmatized and underexplored dimension of academic life, offers critical insights that challenge the limits of purely quantitative analyses. These responses illuminate the emotional labor and personal sacrifices that shape graduate training. It stands as a powerful contribution to ongoing conversations about equity, support, and sustainability in graduate education that cannot be easily dismissed because of the pairing of personal expression with survey data. Moving forward, extended qualitative interviews with graduate students would further illuminate institutional funding practices and constraints, and situate student experiences within their structural contexts.

To preserve anonymity and minimize the potential for miscategorization, the survey did not collect detailed personal demographic information (e.g., ethnicity, gender, or socioeconomic background). While this methodological decision aligns with the ethical standards of the authors, it restricts the ability to analyze how financial burden may differentially affect students across identity categories. The difference between master's and doctoral programs, collapsed in this study, can be further addressed in future research that examines individual program costs and expectations. Master's programs, especially terminal master's programs, are less likely than Ph.D. programs to guarantee multi‐year funding or health coverage, leaving many students to self‐fund or rely on loans (Glover 2019). This research provides an informed foundation that will hopefully elicit such work. Future research can consider other variables like estimates of cost of living, institution types (e.g., R1 institutions, public or private universities, stand‐alone anthropology departments vs. interdisciplinary programs, etc.), and other factors that can impact the funding structures for students. Financial experiences in graduate programs vary considerably across institutional types. For example, students at R1 universities may experience more opportunities for assistantships because of the institution's research focus. Additionally, assuming the larger biological anthropology programs are proportionally represented in the responses, common responses may heavily reflect those individual programs. Parsing out differences based on location in the future could focus on specific cost of living concerns and potential disproportionate local students to some programs, which may take advantage of decreased national competition in the funding offered to those students. While this study intentionally centered student voices, further quantification, particularly regarding institutional characteristics and local economic conditions, would strengthen future analyses and support more nuanced discussions of financial inequity.

In the future, expanded work can include partnership with professional organizations, such as the American Association of Biological Anthropologists, American Academy of Forensic Sciences, American Anthropological Association, the Society for American Archeology to inform the development of new programs that extend beyond current efforts, building on the work of scholars such as Antón et al. (2018) and Malhi et al. (2019). Accordingly, this work should be regarded as a pilot study intended to inform and contribute to broader discussion.

## 6 Conclusion

The financial pressures identified in this study reveal a clear need for reform within graduate programs in bioarchaeology and forensic anthropology. To mitigate the high levels of financial stress and mental strain observed among graduate students, institutions should prioritize initiatives that offer more robust financial support, including livable stipends, comprehensive health coverage, and clear, transparent, equitable funding structures. These findings illustrate the role that financial barriers play in undermining DEI efforts within the field, as students without adequate financial resources are disproportionately affected by current funding limitations.

In response to these challenges, this research advocates for sustainable, student‐centered solutions that reflect the realities faced by graduate students. Key steps include expanding research and professional development funding and fostering transparency around funding and job market expectations. Implementing these changes would advance inclusivity and provide graduate students with the support needed to thrive. If financial instability persists, it risks devaluing the field and stifling the growth and innovation essential to scientific progress.

As the authors of this work, we were driven by our own experiences of financial strain and lack of guidance. We participated in the encouraged unpaid and underpaid labor. We felt the taboo and pressure from institutions and advisors that prevented us from reaching out to peers. This study is fighting that experience and allowing students to express their needs. Students should not have to rely on food pantries and government aid to meet the expectations of their academic program. These results are a shameful but essential look at the state of our field so that these institutions cannot cite ignorance as a defense. We ask departments, professional organizations, and mentors to reconsider the ethics of their expectations for graduate students and make the necessary changes to reduce this financial crisis.

## Author Contributions

**Abigail Elaine Houkes:** conceptualization, writing – original draft, methodology, validation, writing – review and editing, formal analysis, investigation. **Laura Cirillo:** conceptualization, writing – original draft, methodology, validation, writing – review and editing, formal analysis, investigation.

## Funding

This research did not receive any specific grant from funding agencies in the public, commercial, or not‐for‐profit sectors.

## Conflicts of Interest

The authors declare no conflicts of interest.

## Supporting information


**Data S1:** Supporting Information.

## Data Availability

The data that support the findings of this study are available on request from the corresponding author. The data are not publicly available due to privacy or ethical restrictions.
