# Supplementary material for: Financial Barriers to Success: Opening the Discussion of the Financial Burdens and Graduate Student Experiences in Bioarchaeology and Forensic Anthropology
Source: Am J Biol Anthropol. 2025 Dec 12;188(4):e70182. doi: 10.1002/ajpa.70182 (PMC12699365; doi:10.1002/ajpa.70182)
Supplement: Supplementary file 1 — Data S1: Supporting Information. [file AJPA-188-e70182-s001.docx]

**Supplementary Tables**

**Table A.** Questions and response options included in survey.

| **Question** | | **Response Options** | | | |
| --- | --- | --- | --- | --- | --- |
| *Demographic/Education Questions* | | | | | |
| 1. Please indicate what subfield of biological anthropology you are under. If you are multidisciplinary, please answer with your primary subfield. | | Primatology; Paleoanthropology; Molecular Anthropology; Forensic Anthropology; Bioarchaeology; Human Biology; Other (please specify), open-ended field | | | |
| 2. Please indicate what stage of graduate school you are in (pick only one). | | Current Master’s student; Recent Master’s graduate (graduated within the past five years), no Ph.D. education; Current Ph.D. student or candidate, completed a Master’s beforehand; Current Ph.D. student or candidate, has not completed a Master’s beforehand; Rent Ph.D. graduate (graduated within the past five years | | | |
| 3. If you are a current student, how many years into your program are you? | | <1 year; 1 year; 2 years; 3 years; 4 years; 5 years; 6+ years; N/A (Recent graduate) | | | |
| 4. Please indicate which territory on the map represents the location of the graduate institution that you currently attend or most recently attended. | | Visual provided. Territory 1: Western third of US; Territory 2: Central third of US; Territory 3: Midwest and Southern region of US; Territory 4: East coast and New England region of US; International; Attended an institution outside of the United States. | | | |
| *Introduction Questions about Funding Sources* | | | | | |
| 5. Do you, or have you, participated in your school’s Financial Aid program (i.e., scholarships, grants, assistantships, fellowships, tuition reimbursement, federal student loans, and private student loans) within graduate school? | | Yes; No | | | |
| 6. Do you, or have you, received a scholarship, grant, fellowship, or TA/GA/RA’ship at your current or most recent institution? (Excluding tuition waivers) If you have received multiple, answer with the cumulative amount so far within your graduate degree | | Amount less than $1,000; Amount between $1,000 and $5,000; Amount between $5,000 and $10,000; Amount between $10,000 and $50,000; Amount between $50,000 and $90,000; Amount over $90,000 | | | |
| 7. Did you receive funding (grant, fellowship etc.) from an *external* source from your graduate institution? | | No; Yes (Specify number of years funded) | | | |
| 8. Did you receive funding (travel scholarship, material grant, research grant, etc.) from an *internal* source within your graduate institution? | | No; Yes (Specify number of years funded) | | | |
| 9. Within your graduate program, are/were you able to have your tuition waived? | | Yes, all years; Yes, some years; No | | | |
| 10. Did you have a teaching, research, and or graduate assistantship? | | No; Yes (Indicate the average hours per week) | | | |
| 11. Did you have a job within the university other than a teaching, research, or graduate assistantship? | | No; Yes (Indicate the average hours per week) | | | |
| 12. Did you have a job external to the university? | | No; Yes, part time (under 35 hours per week); Yes, full-time (35+ hours per week) | | | |
| *Questions about Financial Strain and Support* | | | | | |
| 13. Have you altered the list of classes you wanted to take to accommodate a job (either internal or external to the university)? | | Yes; No | | | |
| 14. Have you used government aid to afford health care, groceries, or utility bills at any point while enrolled in graduate school? | | Yes; No | | | |
| 15. Have you used a food pantry or other free services within or outside the university to access food or other necessities while enrolled in graduate school? | | Yes; No | | | |
| 16. Were you ever concerned about attending on-campus events (i.e., work, classes, meetings, etc.) due to the cost of transportation (gas for the vehicle, parking pass, paying for daily parking) in graduate school? | | Yes; No | | | |
| 17. Does your graduate program offer a livable wage? A livable wage is defined as the minimum amount of financial assistance (wages) to cover living costs (such as rent, food, utilities, insurance, or anything that you would require) without outside assistance (loans, credit cards, or governmental or family assistance). | | Yes; No | |  |  |
| 18. Please Indicate the degree of your financial expense for the following activities. This could include any fees required (such as program fees, rent, food, course credit, etc.). If you experience changed year-to-year, answer with the average experience.  Internship; Field School; Conference; Research Opportunity. | | I paid for this activity’s expenses completely; I paid for over 50% of this activity’s expenses; I paid for under 50% of the activity’s expenses; I did not pay anything due to the expenses being covered 100%; I was paid for the activity, and I did not have to pay for anything; I did not participate in the activity | |  |  |
| 19. Have you ever felt pressured and or encouraged by a professor/supervisor to participate in the following activities/opportunities to supplement your education or advance your career? This pressure could have been done with or without a discussion of financial support opportunities.  Internship; Field School; Conference; Research Opportunity. | | Yes; No; N/A | | | |
| 20. For the questions below, “more than moderately beneficial” is defined as providing some significant improvement that could not be easily gained outside of this activity. Without this activity, you feel it could be detrimental to your opportunities to gain employment, grants, etc. Please answer for only your graduate school experience and not your undergraduate experience. | |  | |  |  |
| Have you either not applied at all or turned down an *internship* that you think would have been more than just moderately beneficial to your career because of the expense? | | Yes; No; N/A | | | |
| Have you either not applied at all or turned down a *field school* that you think would have been more than just moderately beneficial to your career because of the expense? | | Yes; No; N/A | | | |
| Have you either not applied at all or turned down a *conference opportunity* that you think would have been more than just moderately beneficial to your career because of the expense? | | Yes; No; N/A | | | |
| Have you either not applied at all or turned down a *research opportunity* that you think would have been more than just moderately beneficial to your career because of the expense? | | Yes; No; N/A | | | |
| 21. Did you pay for any of these experiences during graduate school using a credit card, personal loan, or governmental loan in hopes of paying them off in the future? | | Checklist: Internship; Field School; Conference; Professional Attire for Networking Opportunity; Research Opportunity; Tuition; Graduate School Fees; Transportation; Relocation/Moving Costs; Workshop; Certification | | | |
| *Questions about Outlook on Financial Concerns* | | | | | |
| 22. Have your finances during graduate school ever negatively affected your mental health? | | Strongly agree; Agree; Neither agree nor disagree; Disagree; Strongly disagree | | | |
| 23. Have any of the following kept you from discussing finances within the discipline with your *peers*: | | Checklist: Program rules (explicit or common understanding); Explicit request by a supervisor or faculty member; Social discomfort; Other (please specify) | | | |
| 24. Have any of the following kept you from discussing finances within the discipline with your *advisor*: | | Checklist: Program rules (explicit or common understanding); Explicit request by a supervisor or faculty member; Social discomfort; Other (please specify) | | | |
| 25. Current research concerning the financial inequity of graduate students within biological anthropology is sufficient. | | Strongly agree; Agree; Neither agree nor disagree; Disagree; Strongly disagree | | | |
| 26. Professors within my department advocate for financial support of graduate students | | Strongly agree; Agree; Neither agree nor disagree; Disagree; Strongly disagree | | | |
| *Open-ended Questions* | | | | |  |
| 27. Have you ever considered leaving the field of biological anthropology due to the financial cost? | | Yes; No | | | |
|  |  |  | | | |
| If you wish to elaborate on the specifics, please leave your response here. Use of these responses will be paraphrased to remove details of personal experience and protect your privacy. | | Open-ended field | |  |  |
| 28. What actions can graduate institutions take to alleviate the financial burden that graduate students face within biological anthropology? Responses will be paraphrased to remove personal details of your experience and protect your privacy. | | Open-ended field | |  |  |
| 29. Please place any comments, experiences, and/or critiques on financial inequity within biological anthropology in the comment box below. Responses will be paraphrased to remove personal details of your experience and protect your privacy. | | Open-ended field | | | |

**Table B.** Coding scheme for the open-ended response questions.

| Category | Subcategory | Number of Mentions |
| --- | --- | --- |
| Financial Burden | Cost of Living | 14 |
|  | Inadequate Stipends/Funding | 10 |
|  | Student Loans | 7 |
|  | Research Costs | 5 |
| Career Uncertainty | Job Market Saturation | 9 |
|  | Lack of Job Stability | 7 |
|  | Regret About Choosing the Field | 6 |
| Leaving or Considering Leaving | Already Left | 5 |
|  | Considering Leaving | 11 |
|  | Temporary Leave | 3 |
| Familial/External Support | Spousal/Partner Support | 6 |
|  | Parental Support | 5 |
|  | Friends/Communal Support | 2 |
| Mentorship and Guidance | Positive Guidance | 4 |
|  | Lack of Support/Negative Experiences | 4 |
| Diversity and Access | Socioeconomic Barriers | 7 |
|  | Limited Opportunities for Low-Income | 5 |
| Stress/Mental Health | Financial Stress as a Major Contributor | 19 |
|  | Mental Strain from Balancing Work/Studies | 13 |
|  | Uncertainty and Career Anxiety | 12 |
|  | Mental Health Impacts of Leaving the Field | 10 |
|  | Cumulative Effects of Long-Term Pressure | 8 |
|  | Feelings of Isolation and Inequity | 7 |
|  | Coping Mechanisms and Sacrifices | 5 |
|  | Resilience and Persistence | 5 |
| Graduate Program Reform Suggestions | Increase Stipends/Pay a Livable Wage | 12 |
|  | Offer More Grants, Scholarships, and Funding | 9 |
|  | Tuition Waivers and Reduced Fees | 6 |
|  | Improve Housing and Living Support | 5 |
|  | Guarantee Funding for Program Duration | 5 |
|  | Increase Transparency About Funding and Job Prospects | 4 |
|  | Health Insurance and Mental Health Support | 4 |
|  | Limit Enrollment to Only Fundable Students | 3 |
|  | Expand Opportunities for Paid Internships | 3 |
